# Exploring the feasibility of protein phosphatase 1–docking motif-mimetic cell-penetrating peptides for modulating prostate carcinogenesis

**DOI:** 10.1093/jncics/pkaf101

**Published:** 2025-10-14

**Authors:** Renato M Rodrigues, Juliana Felgueiras, Sarah Jones, Vânia Camilo, Bárbara Matos, Carmen Jerónimo, John Howl, Margarida Fardilha

**Affiliations:** Fardilha’s Lab, Department of Medical Sciences, Institute of Biomedicine—iBiMED, University of Aveiro, Aveiro, Portugal; Fardilha’s Lab, Department of Medical Sciences, Institute of Biomedicine—iBiMED, University of Aveiro, Aveiro, Portugal; Cancer Biology and Epigenetics Group—Research Center, Portuguese Oncology Institute of Porto, Porto, Portugal; Research Institute in Healthcare Science, University of Wolverhampton, Wolverhampton, United Kingdom; Cancer Biology and Epigenetics Group—Research Center, Portuguese Oncology Institute of Porto, Porto, Portugal; Fardilha’s Lab, Department of Medical Sciences, Institute of Biomedicine—iBiMED, University of Aveiro, Aveiro, Portugal; Cancer Biology and Epigenetics Group—Research Center, Portuguese Oncology Institute of Porto, Porto, Portugal; Porto Comprehensive Cancer Center, Portuguese Oncology Institute of Porto, Porto, Portugal; Department of Pathology and Molecular Immunology, Institute of Biomedical Sciences Abel Salazar, University of Porto, Porto, Portugal; Research Institute in Healthcare Science, University of Wolverhampton, Wolverhampton, United Kingdom; Fardilha’s Lab, Department of Medical Sciences, Institute of Biomedicine—iBiMED, University of Aveiro, Aveiro, Portugal

## Abstract

**Background:**

Once considered “undruggable,” protein phosphatases are now recognized as potential therapeutic targets. The serine and threonine–protein phosphatase 1 regulates key cellular processes and enhances androgen receptor activity in prostate cancer, even under castration-resistant conditions, suggesting a role in disease progression.

**Methods:**

LNCaP and PC3 cells were treated with peptides mimicking protein phosphatase 1–docking motifs in androgen receptor, alongside known bioportides (MSS1 and mitoparan). Cellular uptake was assessed by confocal microscopy and fluorescence assays. Viability was measured with PrestoBlue, and androgen receptor and Prostate-Specific Antigen expression was analyzed by quantitative reverse transcription–polymerase chain reaction and Western blot.

**Results:**

Androgen receptor sequence contains 3 protein phosphatase 1–docking motifs: KVFF (binding site 1), HVVKW (binding site 2), and KPIYF (binding site 3). Binding site 1 and binding site 2 peptides were modified for better solubility, while binding site 3 was combined with the Tat sequence to enhance cellular uptake. Fluorophore-conjugated peptides successfully entered cells, with androgen receptor–binding site 3 showing the highest internalization in LNCaP cells (*P *= .0495). Treatment with the 3 androgen receptor–binding site peptides individually reduced cell viability in LNCaP and PC3 cells (*P *= .0352 and *P *= .0298, respectively). Combining androgen receptor–binding site peptides statistically reduced cell viability, particularly with all 3 peptides together (LNCaP: 68%, *P *= .0369; PC3: 80%, *P *= .0369). No statistically significant changes in androgen receptor or prostate-specific antigen expression were observed.

**Conclusion:**

Bioportides targeting protein phosphatase 1–docking motifs, especially when combined, decrease prostate cancer cell viability, and additional protein phosphatase 1–interfering peptides such as MSS1 and mitoparan display potent cytotoxic effects. The absence of changes in androgen receptor and prostate-specific antigen expression highlights the need to further investigate their mechanisms of action.

## Introduction

Prostate cancer is the most frequently diagnosed malignancy and a leading cause of cancer-related mortality among men in developed countries.^1^ Although the introduction of targeted therapies in the 1980s clinically improved outcomes across several cancer types,^2^ treatment of advanced prostate cancer remains a clinical challenge. Androgen deprivation therapy, the cornerstone for locally advanced disease, often results in transient responses, with the majority of patients progressing to castration-resistant prostate cancer, a lethal and treatment-refractory stage.^3^ Castration-resistant prostate cancer is frequently driven by sustained androgen receptor activity, including through constitutively active androgen receptor splice variants such as androgen receptor variant 7, which lack the ligand-binding domain and evade classical androgen receptor–targeted therapies.^4^

Recent advances in cancer biology have highlighted protein–protein interactions as key regulators of oncogenic signaling and transcription. These interactions constitute complex intracellular networks that are often dysregulated in prostate cancer.^5^ Notably, serine and threonine-protein phosphatase 1 modulates androgen receptor activity by promoting its nuclear translocation, stability, and transcriptional activity via direct dephosphorylation.^6^^,^^7^ Protein phosphatase 1 has also been shown to regulate the activity of androgen receptor variant 7, further implicating the protein phosphatase 1–androgen receptor interaction as a critical node in androgen receptor signaling and resistance to therapy.^8^ This interaction thus represents a promising therapeutic target in hormone-sensitive and castration-resistant settings.^9^ Although protein phosphatase 1 interacts predominantly with the ligand-binding domain of androgen receptor,^7^ the binding site 1 docking site, located in the hinge region and conserved in androgen receptor variant 7, offers a rational target for disrupting protein phosphatase 1–androgen receptor interactions across isoforms.

However, targeting intracellular protein–protein interactions remains a formidable task. The lack of defined small-molecule binding pockets and the need for cytosolic delivery limit the utility of classical drug design.^10^ In this context, cell-penetrating peptides have emerged as a versatile platform for intracellular delivery of therapeutic agents. Cell-penetrating peptides are short, typically polycationic peptides that cross cellular membranes efficiently and can deliver cargoes ranging from nucleic acids to proteins. Beyond their role as delivery vectors, certain cell-penetrating peptides—termed *bioportides*—display intrinsic biological activity by mimicking functional motifs of endogenous proteins.^11^^,^^12^ These molecules are characterized by rhegnylogical organization, wherein their membrane translocation and bioactivity are structurally separable.^13^ Cell-penetrating peptides also offer an attractive alternative to classical protein–protein interaction inhibitors, as they bypass structural constraints and enable direct intracellular interference with oncogenic interactions such as protein phosphatase 1–androgen receptor.

In the context of prostate cancer, cell-penetrating peptides have already shown promise. For example, a study by Zhang et al.^14^ demonstrated that TAT-conjugated peptides targeting FOXM1 could suppress tumor growth in prostate cancer models. Similarly, cell-penetrating peptides have been used to modulate intracellular kinases and transcriptional regulators relevant to prostate cancer progression, such as c-Myc and survivin. These studies validate the feasibility and potential therapeutic impact of cell-penetrating peptide–based approaches in prostate tumor biology.

Importantly, bioportides such as mitoparan have been shown to induce mitochondrial apoptosis in cancer cells, including prostate-derived lines, by disrupting mitochondrial integrity and activating caspase-mediated death pathways.^15^ These preliminary findings support the rationale for developing bioportides targeting the protein phosphatase 1–androgen receptor axis as a novel therapeutic strategy in prostate cancer.

Despite this progress, bioportides specifically designed to disrupt protein–protein interactions in prostate cancer remain underexplored. A few recent studies have demonstrated the ability of bioportides to modulate androgen receptor signaling or induce apoptosis in prostate-derived cell lines, but a systematic characterization of their uptake, specificity, and molecular impact is lacking.^16^ Given the central role of the protein phosphatase 1–androgen receptor axis in regulating full-length androgen receptor and androgen receptor variant 7 activity, bioportides mimicking protein phosphatase 1–docking motifs in the androgen receptor present a novel and mechanistically grounded strategy to interfere with androgen receptor signaling at a posttranslational level.

In this study, we designed and characterized bioportides derived from protein phosphatase 1–interacting motifs in the androgen receptor. We evaluated their uptake, impact on prostate cancer cell viability, and modulation of androgen receptor and prostate-specific antigen (PSA) expression. Additionally, we tested the effects of MSS1, a known protein phosphatase 1–interfering bioportide, and mitoparan, to further explore the capacity of bioportides to modulate prostate carcinogenesis.^9^ This work contributes to the expanding field of bioactive cell-penetrating peptides and proposes bioportides as innovative tools for the targeted modulation of oncogenic signaling in prostate cancer.

## Methods

### Cell culture

LNCaP (androgen-dependent) and PC3 (castration-resistant) prostate cancer cells were cultured in RPMI 1640 medium with L-glutamine, supplemented with 10% fetal bovine serum and 1% penicillin-streptomycin. For assays, RPMI 1640 without phenol red and supplementation was used. Cells were maintained at 37 °C in a 5% CO_2_ humidified incubator, regularly checked for mycoplasma contamination, and kept at low passage. Viability (>90%) was monitored using trypan blue staining.

### Design, synthesis, and purification of the bioportides

Protein phosphatase 1–docking motifs in androgen receptor were identified using ScanProsite, and cell-penetrating peptide sequences were predicted via CellPPD and CPPpred.^17^^,^^18^ Peptides were synthesized using Fmoc-based solid-phase synthesis on a Liberty Blue synthesizer, following previously established protocols^19^^,^^20^ with minor optimizations to minimize side reactions. Cleavage and fluorescent labeling (tetramethylrhodamine [TAMRA]) were performed under gentle agitation at room temperature, protected from light, following established protocols (AnaTag kit and Thermo Fisher guidelines).^19^^,^^20^ Peptides were subsequently purified by high-performance liquid chromatography and confirmed by mass spectrometry according to established procedures.^12^^,^^19^^,^^20^ Alongside newly designed peptides, we used MSS1 (a refined AKAP4-derived bioportide), its Ala-substituted analogue (AKAP4-BM M), mitoparan, and the cell-penetrating peptides Tat.^9^

### Live-cell confocal microscopy

TAMRA-conjugated peptides were reconstituted in ultrapure water to obtain 1 mM stock solutions, which were filter sterilized (0.2 μm) and stored in aliquots at -20 °C until used. Peptide translocation into cells was assessed by live-cell confocal microscopy as previously described.^12^^,^^20^ Briefly, cells were grown in standard growth conditions to 60%-70% confluence in 35-mm sterile glass base dishes. Peptides were diluted in RPMI-1640 medium without phenol red and added to cells, previously washed in RPMI-1640 medium without phenol red, to a final concentration of 5 μM. Cells were incubated for 1 hour at 37 °C, protected from light, in a humidified incubator with 5% CO_2_ atmosphere. Following the incubation time, cells were gently washed in RPMI-1640 medium without phenol red and left in 2-mL medium for microscopic examination. Untreated cells and cells incubated with TAMRA-conjugated Tat were included as background reading and positive control, respectively. Images were acquired in a Zeiss LSM 510M confocal microscope (CarlZeiss Microimaging GmbH, Germany) equipped with an environmental chamber for adequate temperature and CO_2_ control of living cells.

### Quantitative uptake of fluorescent bioportides

Quantitative analysis of peptide translocation was performed using TAMRA-conjugated peptides. Cells were grown in standard growth conditions to 80% confluence in 6-well plates. Cells were then washed in RPMI-1640 medium without phenol red and incubated with 5 μM TAMRA-labeled peptides for 1 hour, as indicated for live-cell confocal microscopy. After cells were collected as previously described with a few modifications,^12^^,^^20^ they were washed 4 times with HBSS (phenol red-free, Sigma-Aldrich, USA), detached with 10% trypsin (phenol red-free) at 37 °C, and centrifuged at 6000 rpm for 5 minutes at 4 °C. Pellets were resuspended in 300 μL 0.1 M NaOH and lysed overnight at -20 °C. Fluorescence (λAbs 544 nm/λEm 590 nm) was measured in an Infinite 200 PRO reader (Tecan, Switzerland) using 250 μL lysate in a 96-well black plate. Each condition had 3 replicates, repeated in 3 independent experiments.

### Cell viability

One mM peptide stocks were prepared as for fluorescent versions. Cells were grown to 60%-70% confluence in 96-well plates and treated with 1-20 μM peptides in RPMI-1640 (phenol red-free). After 24 or 48 hours at 37 °C, 5% CO_2_, PrestoBlue (1/10 dilution) was added for 30 minutes. Fluorescence (λAbs 560 nm/λEm 600 nm) was measured using an Infinite 200 PRO reader (Tecan). Each condition had 3-5 replicates, repeated in 3 independent experiments.

### Real-time polymerase chain reaction

Cells were incubated with 10 µM androgen receptor binding site 1, androgen receptor binding site 2, androgen receptor binding site 3, or their combination in RPMI-1640 (phenol red-free) for 24 hours at 37 °C, 5% CO_2_. RNA was extracted with TRIzol (Invitrogen, USA) and converted to cDNA using the RevertAid RT Kit (Thermo Fisher, USA). Androgen receptor and PSA expression was quantified by quantitative polymerase chain reaction (PCR) using NZYSpeedy quantitative PCR Green Master Mix (NZYTech, Portugal), normalized to GUSB, and analyzed on a 7500 Real-Time PCR system (Applied Biosystems, USA) via the relative standard curve method. The experiment was performed in duplicate, with triplicate quantitative PCR reactions per sample.

### Protein quantification

Protein quantification was performed using the Pierce BCA Protein Assay Kit.

### Western blot analysis

Cells were lysed on ice using lysis buffer (Beyotime, Shanghai, China, P0013) and centrifuged at 12 000 rpm at 4 °C for 15 minutes. A 5× loading buffer was added, and samples were boiled for 10 minutes. Proteins were separated by 12% sodium dodecyl sulfate–polyacrylamide gel electrophoresis (SDS-PAGE) and transferred onto polyvinylidene difluoride (PVDF) membranes. Transfer efficiency was confirmed using Ponceau staining, also used as a loading control. After blocking with 5% fat-free milk in Tris-buffered saline with Tween 20 (TBS-T), membranes were incubated with primary antibodies (anti-androgen receptor and anti-beta-tubulin, 1:1000) for 1 hour or overnight at 4 °C, followed by incubation with secondary antibodies for 1 hour at room temperature. Detection was performed using an Odyssey infrared imaging system (Li-COR, Biosciences, USA).

### Data and statistical analysis

Image analysis was done using Bio-Rad ImageLab (v6.1) after initial use of QuantityOne. Data were processed in Excel, and graphs were created in RStudio (v2023.03.1 + 446). Statistical analysis was performed in SPSS (v25.0) using the Kruskal–Wallis and Mann–Whitney *U* tests (α = 0.05).

## Results

### Synthesis of linear peptides derived from the protein phosphatase 1–docking motifs in androgen receptor’s primary sequence

**Figure 1.**
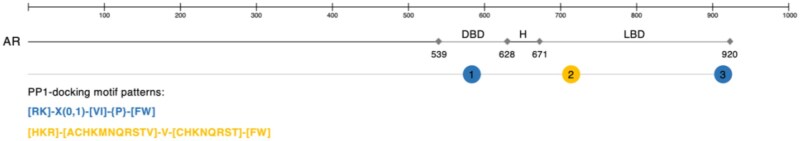
Partial androgen receptor’s primary sequences and protein phosphatase 1 (PP1)–docking motifs. Androgen receptor’s primary sequence contains 3 PP1-docking motifs (**numbered circles**): 1 localized at the DNA-binding domain (DBD) and 2 at the ligand-binding domain (LBD). These 2 regions are separated by a small hinge region (H).

Androgen receptor’s amino acid sequence contains 3 protein phosphatase 1–docking motifs, which are all RVxF sequences: ^581^KVFF^584^, ^715^HVVKW^719^, and ^913^KPIYF^917^ (Figure 1). Whereas the binding site 1 is localized at the DNA-binding domain and is conserved among all androgen receptor isoforms, binding site 2 and 3 are localized at the ligand-binding domain and are only shared by androgen receptor full length (the canonical isoform) and androgen receptor variant 45 (Figure S1). These sequences and additional flanking residues were analyzed using cell-penetrating peptide prediction databases, and peptides’ solubility was calculated as a first approach to their design. The first 2 sequences were identified as candidate rhegnylogic bioportides, and only a small amino acid change was applied in each of them to improve solubility (Table 1 and Table S1). Because of its predicted poor performance as a cell-penetrating peptide (Table S1), the third sequence was coupled to the Tat sequence (Table 1 and Table S1), a well-known and efficient cell-penetrating peptide.^21^

**Table 1.** Androgen receptor–binding site peptides’ sequences

Peptide	Sequence^a^	Length (amino acid)	Mass (g/mol)
Androgen receptor–binding site 1^b^	GSC**KVFF**KRAAKGKQK-NH_2_	16	1782.17
Androgen receptor–binding site 2^c^	RQLV**HVVKW**AKKL-NH_2_	13	1605.01
Androgen receptor–binding site 3	KV**KPIYF**HTGRKKRRQRRRPPQ-NH_2_	22	2832.40

a Protein phosphatase 1–docking motifs are in bold. Amino acid changes from the primary amino acid sequence are underlined. Tat sequence is within the rectangle.

b Amino acid change: E-K.

c Amino acid change: A-K.

### Translocation of the androgen receptor–binding site peptides into prostate cancer cells

**Figure 2.**
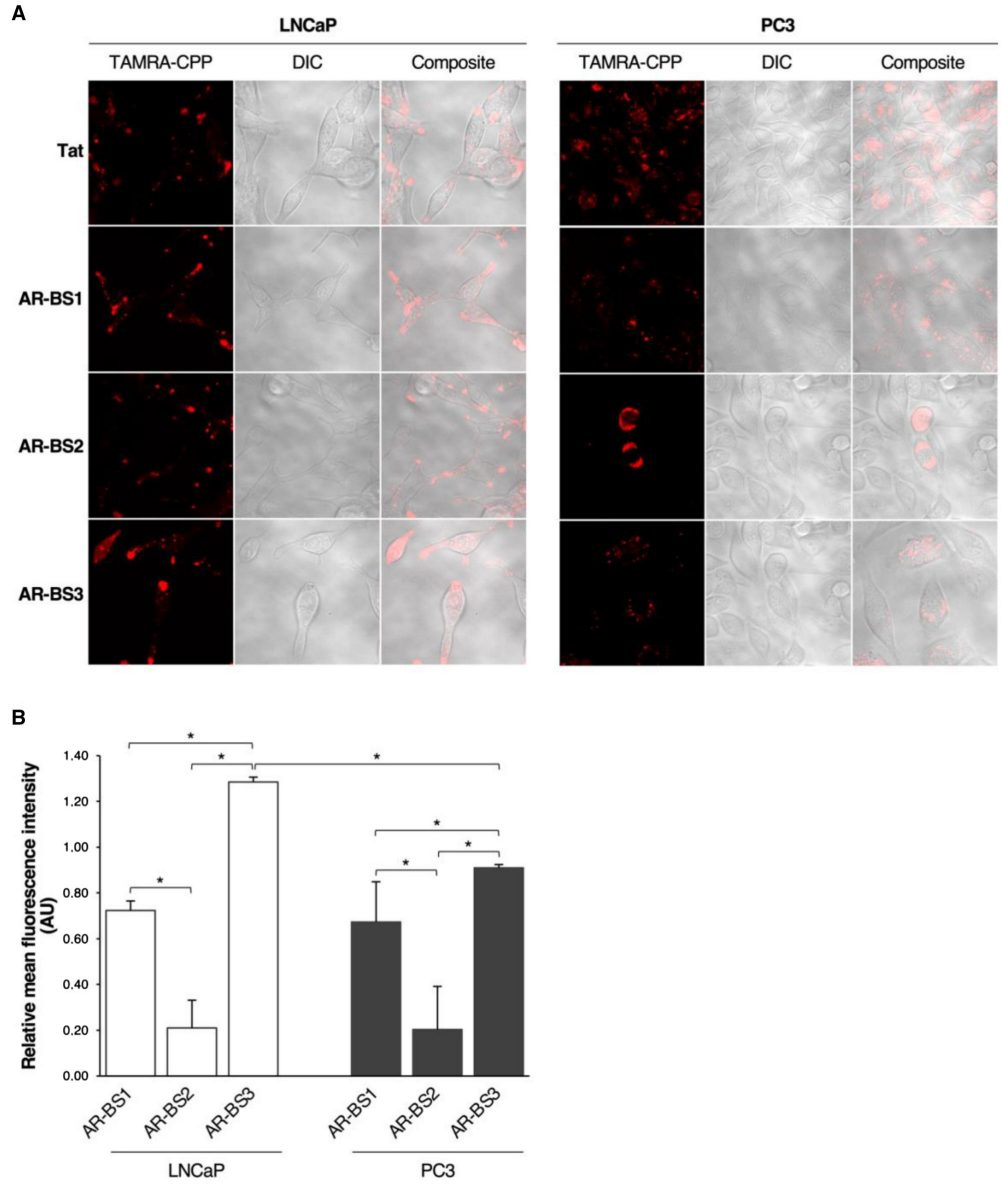
Translocation of AR-BS peptides into prostate cancer cells. LNCaP and PC3 cells were treated with TAMRA-labeled AR-BS peptides (5 μM) and incubated for 1 hour at 37°C in a humidified incubator with 5% CO_2_ atmosphere. Cells incubated with TAMRA-Tat CPP under the same conditions were used as a positive control for CPP internalization. Cells were then washed and visualized by live-cell confocal microscopy (magnification 1000×) (**A**) or lysed for internal fluorescence intensity measurements (**B**). Normalized data are expressed as the ratio of mean (SD) fluorescence (minus background) to Tat fluorescence (minus background) from 3 independent experiments performed in triplicate. Comparisons between peptides’ internalization were assessed using the Mann–Whitney *U* statistical test. *P < .05. Abbreviations: AR-BS = androgen receptor–binding site; AU = arbitrary units; CPP = cell-penetrating peptide; DIC = differential interference contrast.

Fluorophore-conjugated versions of the androgen receptor–binding site peptides were employed to visualize their intracellular distribution by live-cell confocal microscopy and to assess their internalization quantitatively. The 3 peptides demonstrated cellular penetration after 1 hour of incubation, mostly showing a vesicular distribution throughout the cytoplasm (Figure 2A). Punctual localization within the nucleus and nucleolus was found particularly for androgen receptor–binding site 3 (Figure 2A). Quantitative uptake experiments showed that the peptides were differentially internalized by LNCaP (*P *= .0273) and PC3 cells (*P *= .0273). Androgen receptor–binding site 3 was the most efficiently internalized among the 3, with a translocation efficacy of 0.91 in PC3 cells and 1.29 in LNCaP cells (*P *= .0495), performing better than Tat cell-penetrating peptide in LNCaP cells (Figure 2B). Androgen receptor–binding site 1 was moderately cell penetrating, with an efficacy index of 0.72 and 0.67 in LNCaP and PC3 cells, respectively (Figure 2B). Androgen receptor–binding site 2 showed less efficient cell-penetrating properties, with a translocation efficacy of approximately 0.20 in both cell lines (Figure 2B). All fluorescence measurements were normalized to total cell number and background signal.

### Effect of androgen receptor–binding site peptides, MSS1, and mitoparan, alone or in combination, on prostate cancer cells’ viability

An exploratory study was performed by incubating cells with different concentrations of androgen receptor–binding site peptides (1, 3, 5, 10, and 20 μM) for 24 hours. Treatment with 5, 10, or 20 μM of each androgen receptor–binding site peptide for 24 hours did not produce a dose-dependent effect (Table S2). Based on these results, subsequent analyses were performed using 10 μM of androgen receptor–binding site 1, androgen receptor–binding site 2, androgen receptor–binding site 3, AKAP4 BM M, MSS1, or mitoparan for 24 hours.

**Figure 3.**
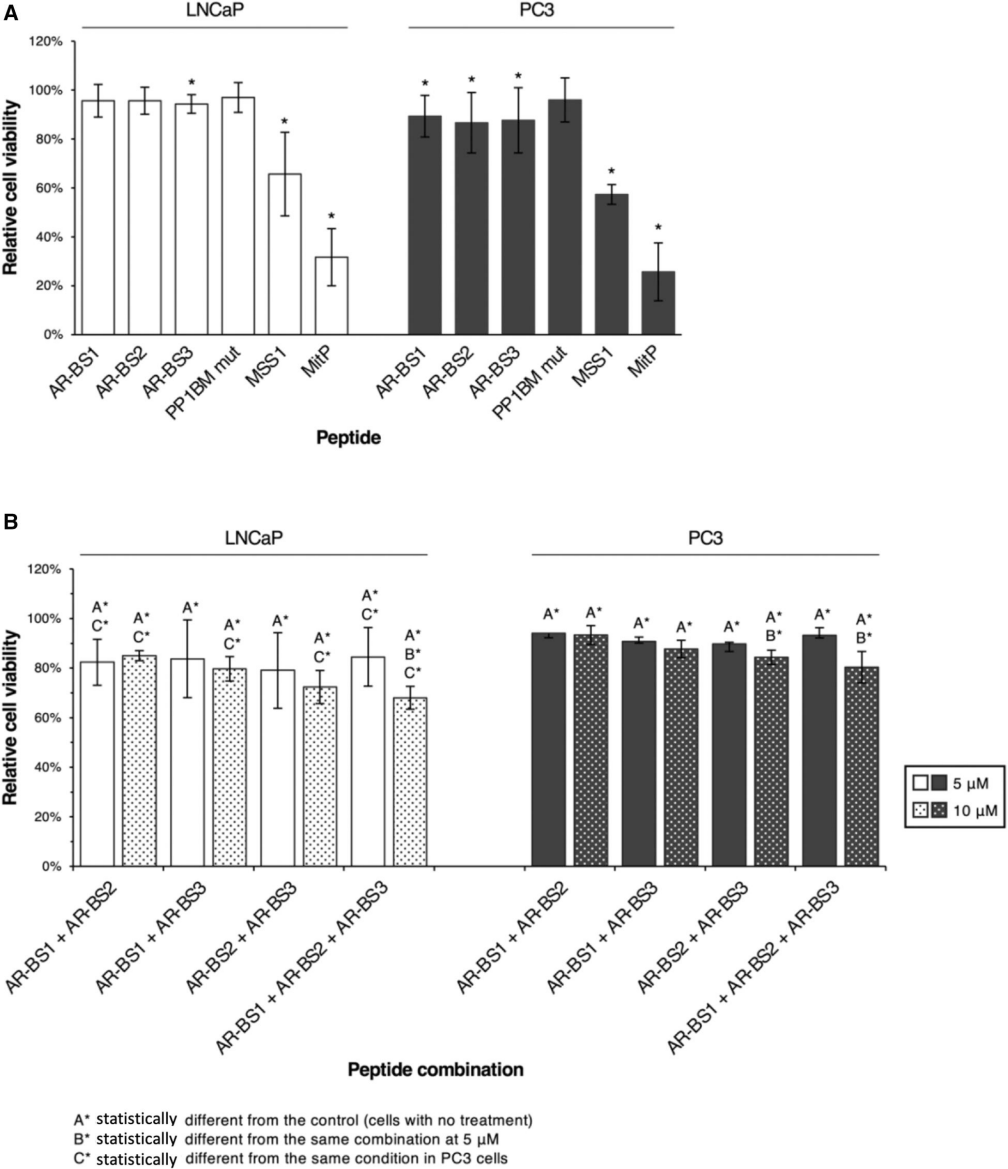
Synergistic reduction of prostate cancer cell viability by combined AR-BS peptides. **A**) LNCaP and PC3 cells were treated with AR-BS1, AR-BS2, AR-BS3, AKAP4 BM M, MSS1, or mitP (10 μM) for 24 hours. **B**) Cells were treated with combinations of AR-BS peptides (5 or 10 μM of each peptide) for 24 hours to assess potential synergistic effects. Cell viability was evaluated using the PrestoBlue Cell Viability Reagent according to the manufacturer’s instructions. Percentage cell viability was calculated as the ratio between treatment and control conditions (cells without treatment). Results are expressed as mean (SD) percentage from 3 independent experiments with 5 replicates per condition. Statistical comparisons were performed using the Mann–Whitney *U* test. *P < .05. Abbreviations: AR-BS = androgen receptor–binding site; mitP = mitoparan.

Differences in cell viability were observed for LNCaP (*P *= .0352) and PC3 (*P *= .0298) cells. In LNCaP cells, androgen receptor–binding site 3 (mean = 96%, *P *= .0369), MSS1 (mean = 66%, *P *= .0369), and mitoparan (mean = 32%, *P *= .0369) statistically reduced viability compared with untreated controls (Figure 3A). In PC3 cells, all 3 androgen receptor–binding site peptides induced a similar reduction (androgen receptor–binding site 1, mean = 89%; androgen receptor–binding site 2, mean = 87%; and androgen receptor–binding site 3, mean = 88%; *P *= .0369), whereas MSS1 (mean = 57%, *P *= .0339) and mitoparan (mean = 26%, *P *= .0369) had a more pronounced effect (Figure 3A). The mutant AKAP4 peptide lacking the protein phosphatase 1–docking motif (AKAP4 BM M) had no effect in either cell line (Figure 3A). These results indicate that mitoparan and MSS1 have the strongest impact on prostate cancer cell viability, with androgen receptor–binding site peptides showing a minor effect when used individually.

To assess potential synergetic effects, cells were treated with combinations of androgen receptor–binding site peptides (5 or 10 μM each) for 24 hours. All combinations statistically decreased viability in LNCaP and PC3 cells compared with controls (Figure 3B). Dose-dependent reductions were observed only for androgen receptor–binding site 2 + androgen receptor–binding site 3 in PC3 cells (*P *= .0463) and for the triple combination (androgen receptor–binding site 1 + androgen receptor–binding site 2 + androgen receptor–binding site 3) in LNCaP (*P *= .0495) and PC3 (*P *= .0463) cells (Figure 3B). Synergetic effects were more pronounced in LNCaP cells, with the triple combination at 10 μM each, producing the largest decrease in viability (mean = 68% for LNCaP, and mean = 80% for PC3; *P *= .0369) (Figure 3B). Overall, these findings support a synergetic action of androgen receptor–binding site peptides in reducing prostate cancer cell viability, particularly when all 3 are combined.

### Effect of androgen receptor–binding site peptides on androgen receptor and PSA transcriptional levels

**Fig 4.**
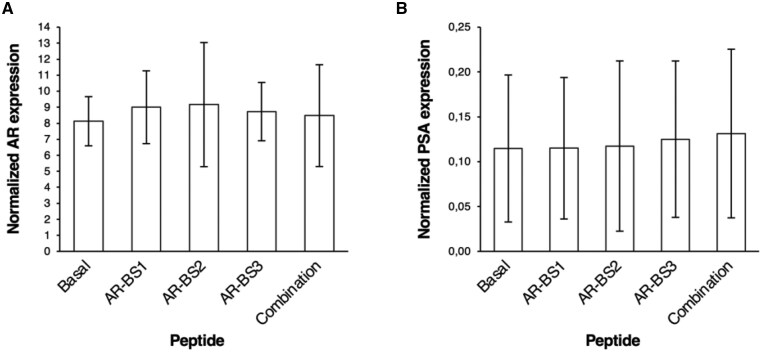
AR and PSA transcriptional levels in response to treatment with AR-BS peptides. LNCaP cells were treated with 10 μM of AR-BS1, AR-BS2, or AR-BS3, or a combination of the 3 (10 μM each) for 24 hours. Total RNA was extracted, and real-time polymerase chain reaction analysis was performed to quantify the levels of AR (**A**) and PSA (**B**). Expression values were normalized to the levels of the BGUS housekeeping gene. Each condition was performed in triplicate, and the experiment was repeated twice. Abbreviations: AR = androgen receptor; BS = binding site; PSA = prostate-specific antigen.

Because protein phosphatase 1 was reported to increase androgen receptor expression and transcriptional activity, we went further to assess the transcriptional levels of androgen receptor and PSA in LNCaP cells in response to treatment with each androgen receptor–binding site peptide (10 μM) or the combination of the 3 (10 μM each). No clinically differences were observed in the levels of either androgen receptor or PSA after each treatment (Figure 4).

## Discussion

Cell-penetrating peptides have emerged as valuable tools for targeting intracellular protein–protein interactions in cancer.^7^ Androgen receptor–mediated signaling plays a crucial role in prostate cancer progression, making it a key therapeutic target.^8^ Protein phosphatase 1 is a positive regulator of androgen receptor expression and activity,^10^^,^^11^ interacting directly with androgen receptor, although the precise binding mechanism remains unclear.

Bioinformatics analysis identified 3 protein phosphatase 1–docking motifs in androgen receptor (Figure 1). To explore their role, we designed and synthesized 3 bioportides (androgen receptor–binding site 1, androgen receptor–binding site 2, and androgen receptor–binding site 3) mimicking these motifs. All peptides were internalized by androgen-dependent and castration-resistant prostate cancer cells, albeit with varying efficiencies (Figure 2). Although cell-penetrating peptides generally enter cells via membrane translocation or endocytosis, intracellular factors such as proteolysis influence their accumulation.^22^^,^^23^ Androgen receptor–binding site peptides exhibited vesicular distribution (Figure 2A), though androgen receptor–binding site 2 had a lower intracellular concentration (Figure 2B), contradicting its high predicted uptake (Table S1). This may result from intracellular processing, as androgen receptor–binding site 2 features a distinct RVxF motif with higher specificity but lower sensitivity for protein phosphatase 1 binding.^24^

Individually, androgen receptor–binding sites 1, 2, and 3 reduced cell viability to a similar extent (Figure 3A). However, none of the peptides altered androgen receptor or PSA transcription (Figure 4). This disconnect suggests that their cytotoxicity may not derive exclusively from disruption of androgen receptor and protein phosphatase 1 interaction but rather from broader cellular stress responses or off-target effects. Furthermore, as PC3 cells lack detectable androgen receptor expression, the contribution of androgen receptor–dependent pathways remains uncertain. This limitation highlights the need for validation in androgen receptor–positive models such as LNCaP.

Interestingly, combining androgen receptor–binding site peptides produced a stronger reduction in viability (Figure 3B). One possible explanation is a synergistic effect in which simultaneous engagement of multiple protein phosphatase 1–binding motifs more effectively perturbs protein phosphatase 1–regulated complexes. Nevertheless, the absence of changes in androgen receptor or PSA expression indicates that even this combined strategy may not directly destabilize androgen receptor signaling. An alternative possibility is that the peptides interfere with other protein phosphatase 1 substrates or cellular pathways critical for survival. Thus, although the synergistic effect is promising, mechanistic clarification is required.

The androgen receptor and protein phosphatase 1 interaction plays a key role in androgen receptor stability, as protein phosphatase 1 dephosphorylates ARSer650 in the ligand-binding domain, preventing androgen receptor degradation and enhancing transcriptional activity.^10^ The lack of effect of androgen receptor–binding site peptides on androgen receptor or PSA levels does not exclude posttranslational modulation, which was not assessed here. Likewise, no direct evidence was obtained that the peptides competitively inhibit protein phosphatase 1–androgen receptor binding. These questions remain important targets for future experiments.

Beyond androgen receptor–targeting peptides, MSS1—an optimized AKAP4-mimetic peptide developed to modulate sperm motility—also decreased prostate cancer cell viability (Figure 3A). AKAP4, a cancer and testis antigen, was detected in PC3 cells, though protein phosphatase 1 γ2 was absent, suggesting MSS1 may target other protein phosphatase 1 isoforms.^25^ Additionally, Mitoparan (MitP), a mitochondriotoxic bioportide, exhibited cytotoxic effects in androgen-dependent and castration-resistant prostate cancer cells (Figure 3A), supporting its potential role in prostate carcinogenesis.^23^

Some limitations should be noted. First, no experiments were conducted in nontumorigenic prostate cells, which precludes assessment of selectivity and raises the possibility of unspecific toxicity. Second, the mechanism underlying the observed loss of viability remains undefined. Third, biochemical characterization of peptide–protein phosphatase 1 or peptide–androgen receptor interactions is lacking. Addressing these gaps will be critical to advancing this strategy toward therapeutic relevance.

In conclusion, androgen receptor and protein phosphatase 1 interaction remains a promising target in prostate cancer therapy. Although the present study shows that androgen receptor–non-specific (NS) peptides—particularly when combined—reduce prostate cancer cell viability, the absence of detectable effects on androgen receptor or PSA expression highlights mechanistic uncertainties. Future studies should (1) verify androgen receptor–dependent effects in androgen receptor–positive cell models, (2) confirm direct disruption of protein phosphatase 1–androgen receptor binding, (3) assess posttranslational modifications of androgen receptor, and (4) evaluate specificity in nontumorigenic cells. Optimization of peptide stability and efficacy will also be required to enhance their translational potential.

## Supplementary Material

pkaf101_Supplementary_Data

## Data Availability

The data underlying this article will be made available in a public repository upon publication. A DOI or permanent link will be provided at that time. Data will be shared in accordance with the FAIR principles and will be accessible for reuse.
