# Supplementary material for: Exploring the feasibility of protein phosphatase 1–docking motif-mimetic cell-penetrating peptides for modulating prostate carcinogenesis
Source: JNCI Cancer Spectr. 2025 Oct 14;9(6):pkaf101. doi: 10.1093/jncics/pkaf101 (PMC12620000; doi:10.1093/jncics/pkaf101)
Supplement: pkaf101_Supplementary_Data [file pkaf101_supplementary_data.zip › Supplementary data.docx]

Supplementary data


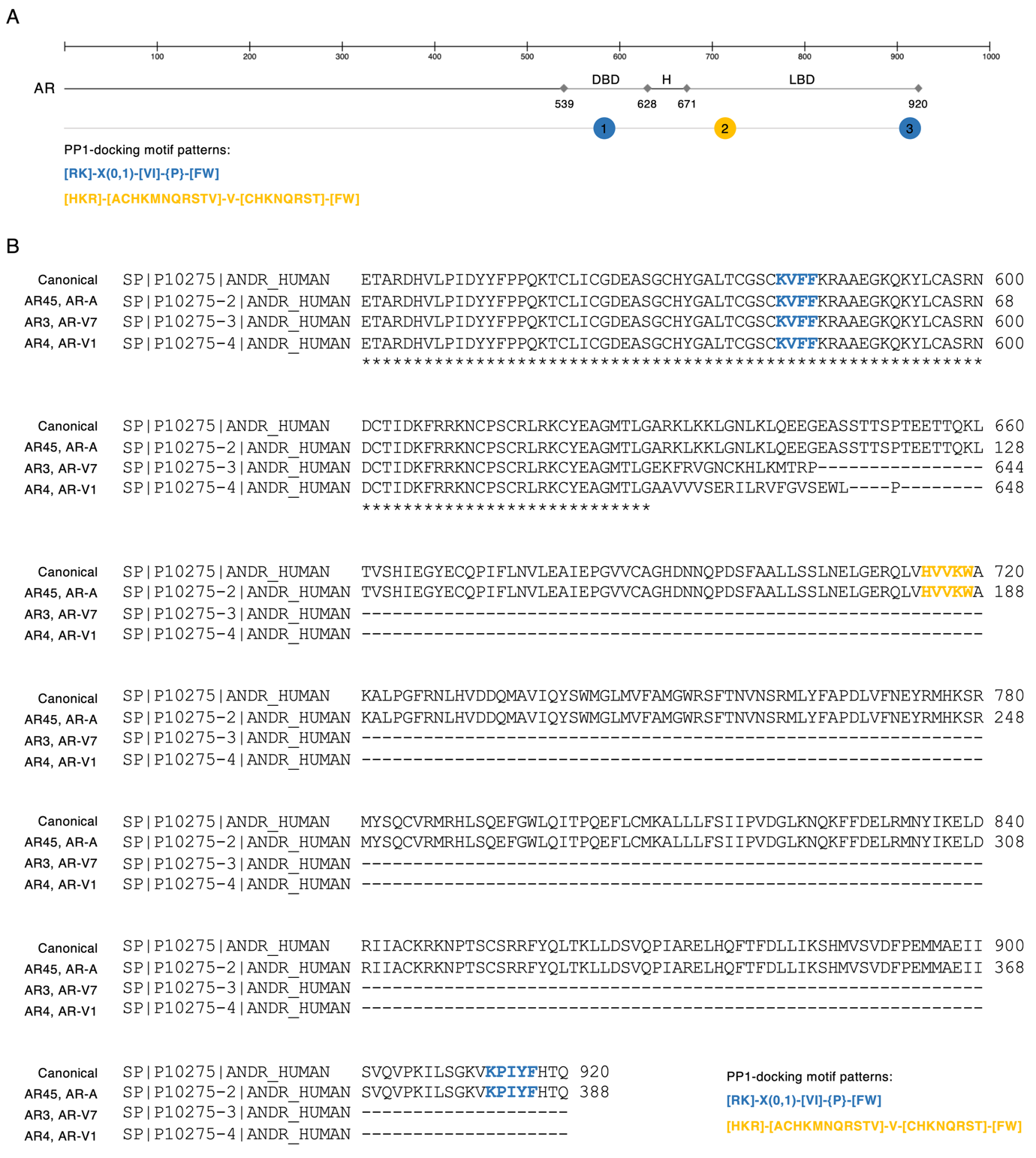


**Fig. S1.** Alignment of partial AR isoform primary sequences showing conservation of PP1 docking motifs across isoforms. The alignment highlights the conserved PP1 docking motifs, which are colour-coded according to their consensus pattern, as indicated in the figure.

Table S1. Cell-penetrating peptides prediction based on the PP1‑docking motifs in AR's primary sequence.

| **Peptide sequence** | **Score** | **Charge** | **Molecular weight** |
| --- | --- | --- | --- |
| Binding site 1 |  |  |  |
| GSC**KVFF**KRAA | 0.290 | 3.00 | 1213.60 |
| GSC**KVFF**KRAAEGKQK | 0.421 | 4.00 | 1784.33 |
| GSC**KVFF**KRAAKGKQK | 0.570 | 6.00 | 1783.39 |
| Binding site 2 |  |  |  |
| RQLV**HVVKW**AKAL | 0.750 | 3.50 | 1548.11 |
| RQLV**HVVKW**AKKL | 0.793 | 4.50 | 1605.21 |
| Binding site 3 |  |  |  |
| SGKV**KPIYF**HTQ | 0.106 | 2.50 | 1404.81 |
| SGKV**KPIYF**HTGRKKRRQRRRPPQ | 0.736 | 10.50 | 2977.87 |
| The potential peptides were selected from the analysis of AR's primary sequence. PP1‑docking motifs (bold) and flaking residues were analyzed through CPP prediction databases and changes were introduced to improve their scores. Scores were obtained from CPPpred (http://bioware.ucd.ie/cpppred (Holton, Pollastri, Shields, & Mooney, 2013)) and are interpreted as follows: 0-0.5, the peptide is very unlikely to be cell‑penetrating; 0.5-1.0, the peptide is predicted to be cell‑penetrating (the closer to 1.0 the more confident that the peptide will be cell‑penetrating). Charge and molecular weight were calculated using CellPPD (http://crdd.osdd.net/raghava/cellppd/ (A Gautam et al., 2013; Ankur Gautam, Chaudhary, Kumar, & Raghava, 2015)). AR, androgen receptor; PP1, serine/threonine‑protein phosphatase PP1. | | | |

Table S2. Cell viability in response to treatment with different concentrations of AR-BS peptides for 24 h.

|  |  | **LNCaP** | | |  | **PC3** | | |
| --- | --- | --- | --- | --- | --- | --- | --- | --- |
|  |  | 5 μM | 10 μM | 20 μM |  | 5 μM | 10 μM | 20 μM |
| **AR-BS1** | Mean | 97% | 96% | 94% |  | 89% | 89% | 81% |
|  | SD | 0,08 | 0,07 | 0,05 |  | 0,09 | 0,09 | 0,12 |
| **AR-BS2** | Mean | 95% | 96% | 83% |  | 93% | 87% | 81% |
|  | SD | 0,09 | 0,06 | 0,18 |  | 0,08 | 0,12 | 0,17 |
| **AR-BS3** | Mean | 91% | 94% | 99% |  | 79% | 88% | 84% |
|  | SD | 0,11 | 0,04 | 0,07 |  | 0,16 | 0,13 | 0,10 |

Percentage cell viability was calculated from the ratio between treatment condition and control condition (cells with no treatment). Results are expressed as mean ± SD from three independent experiments with five replicates per condition.

Gautam, A, Chaudhary, K., Kumar, R., Sharma, A., Kapoor, P., & Tyagi, A. (2013). Open source drug discovery consortium. Raghava GPS: In silico approaches for designing highly effective cell penetrating peptides. *J Transl Med*, *11*, 1–12. https://doi.org/10.1186/1479-5876-11-74

Gautam, Ankur, Chaudhary, K., Kumar, R., & Raghava, G. P. S. (2015). Computer-Aided Virtual Screening and Designing of Cell-Penetrating Peptides. *Methods in Molecular Biology (Clifton, N.J.)*, *1324*, 59–69. https://doi.org/10.1007/978-1-4939-2806-4_4

Holton, T. A., Pollastri, G., Shields, D. C., & Mooney, C. (2013). CPPpred: prediction of cell penetrating peptides. *Bioinformatics (Oxford, England)*, *29*(23), 3094–3096. https://doi.org/10.1093/bioinformatics/btt518
